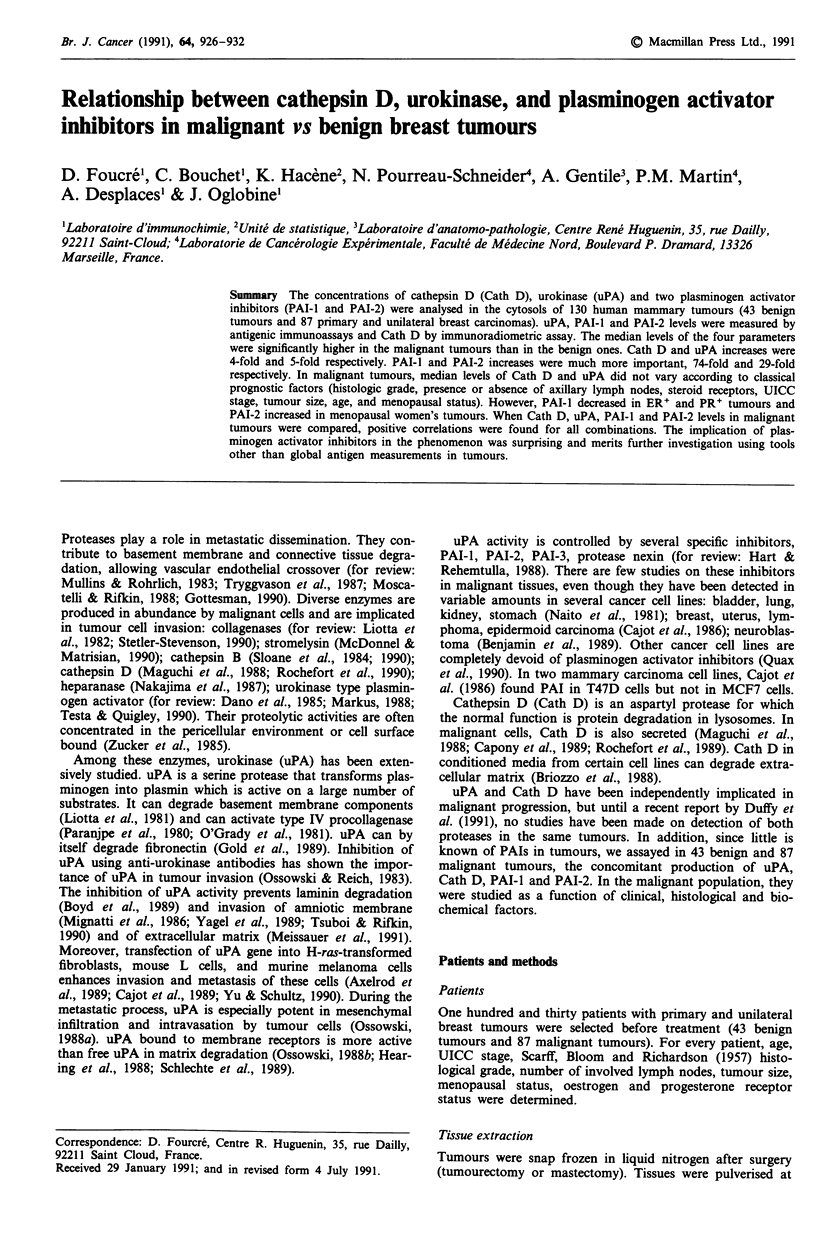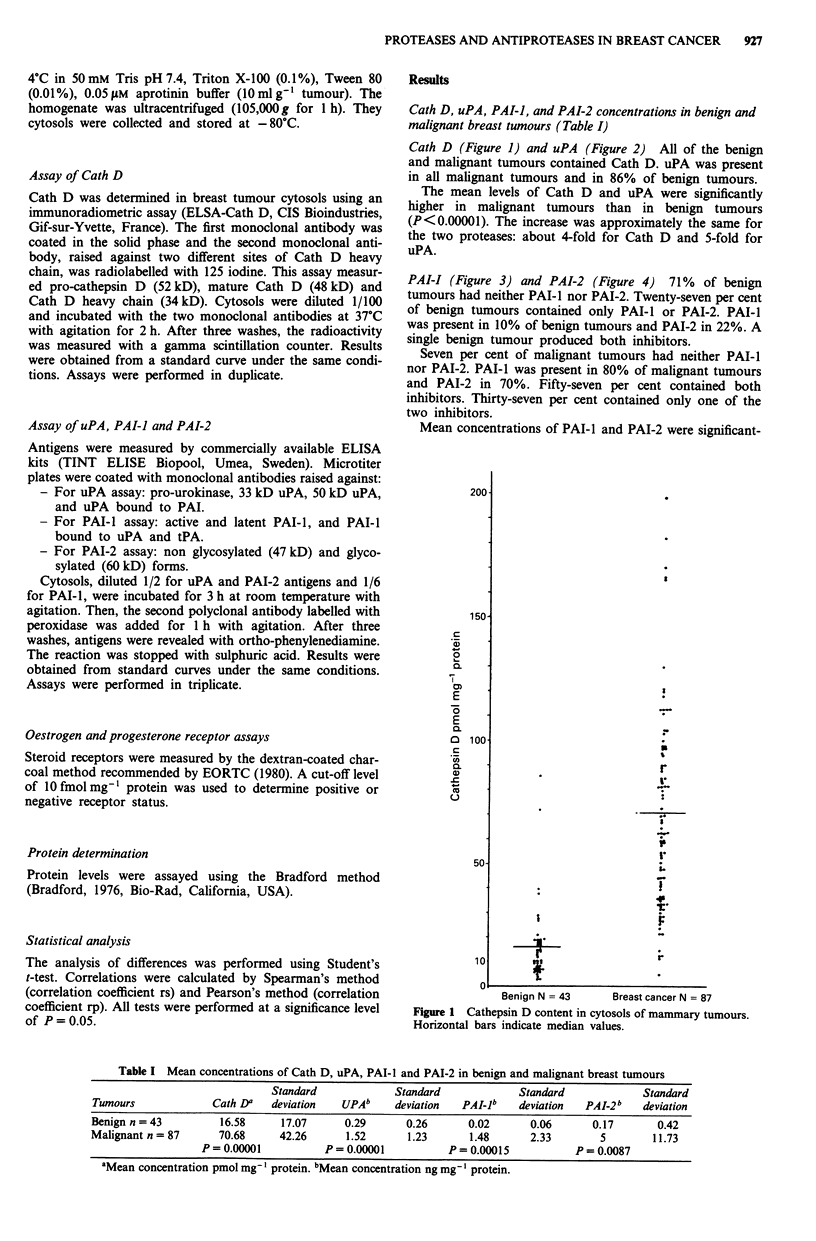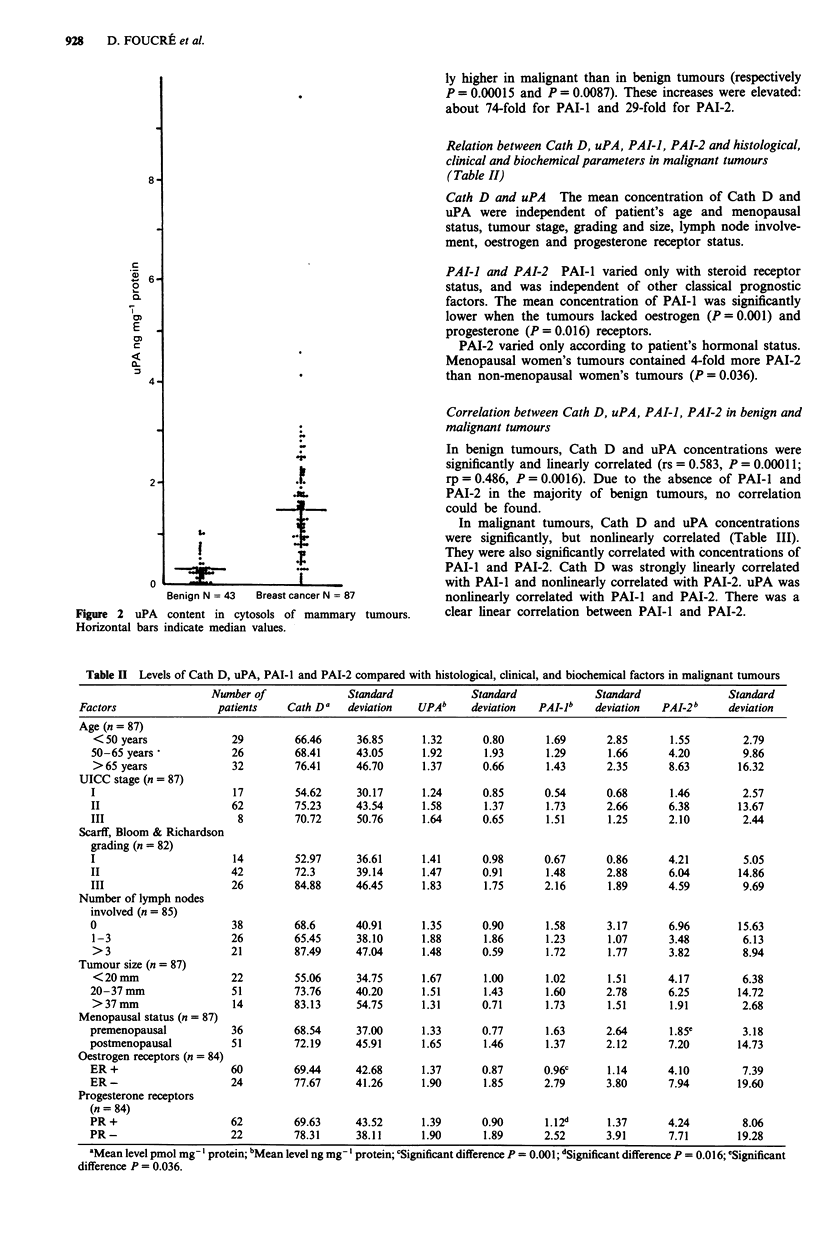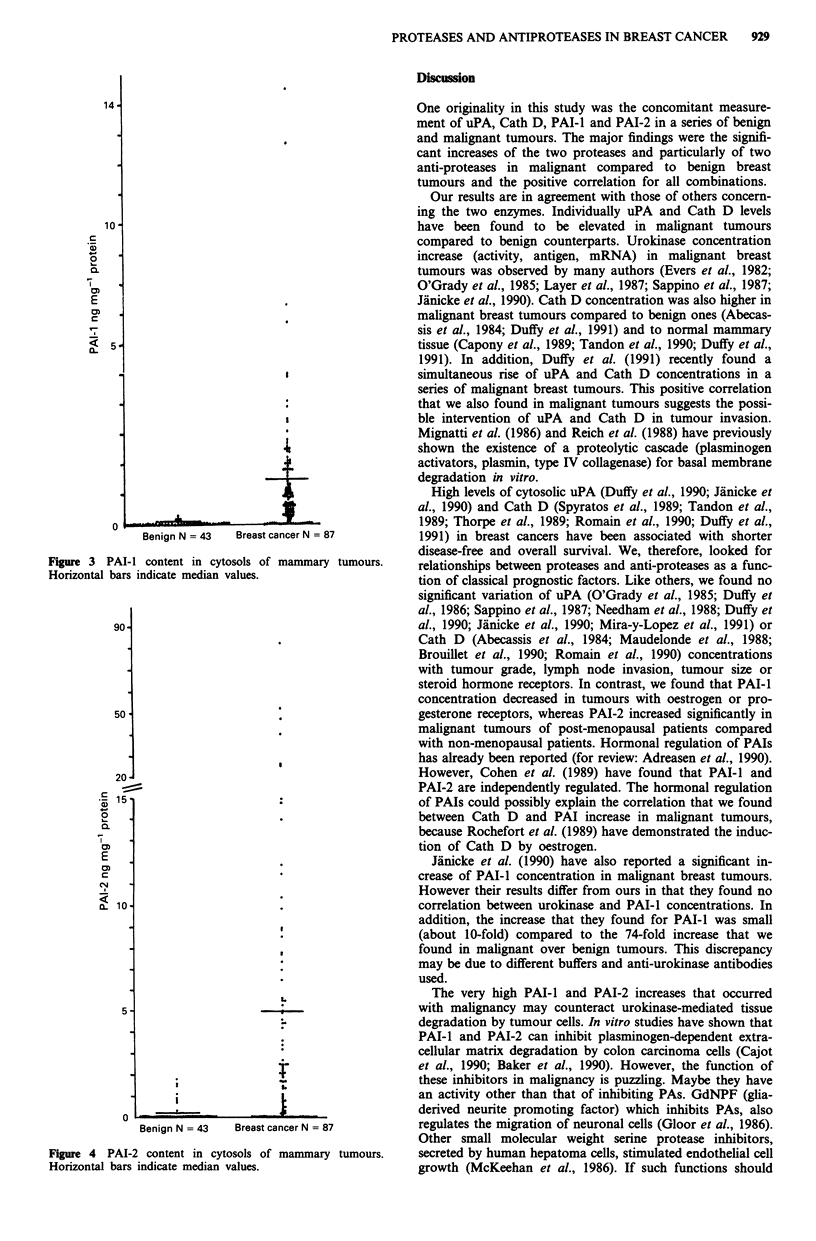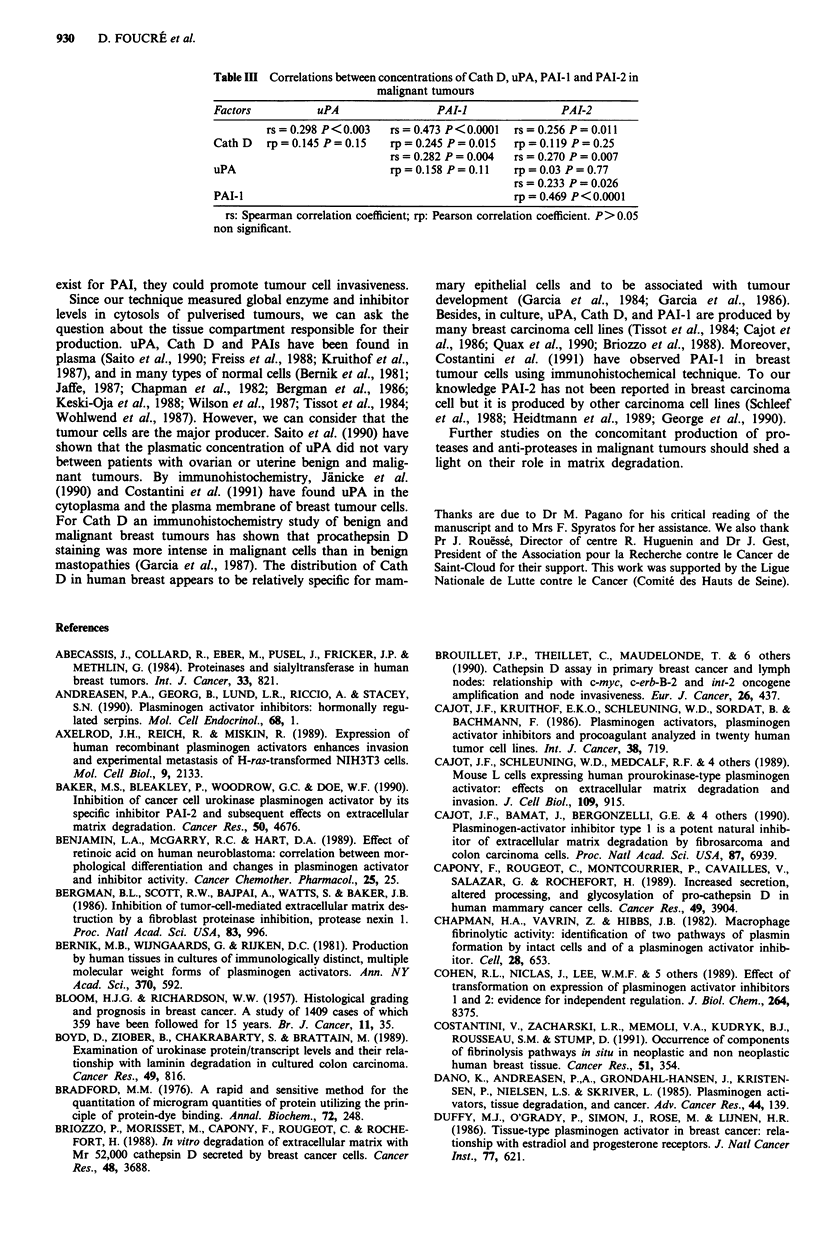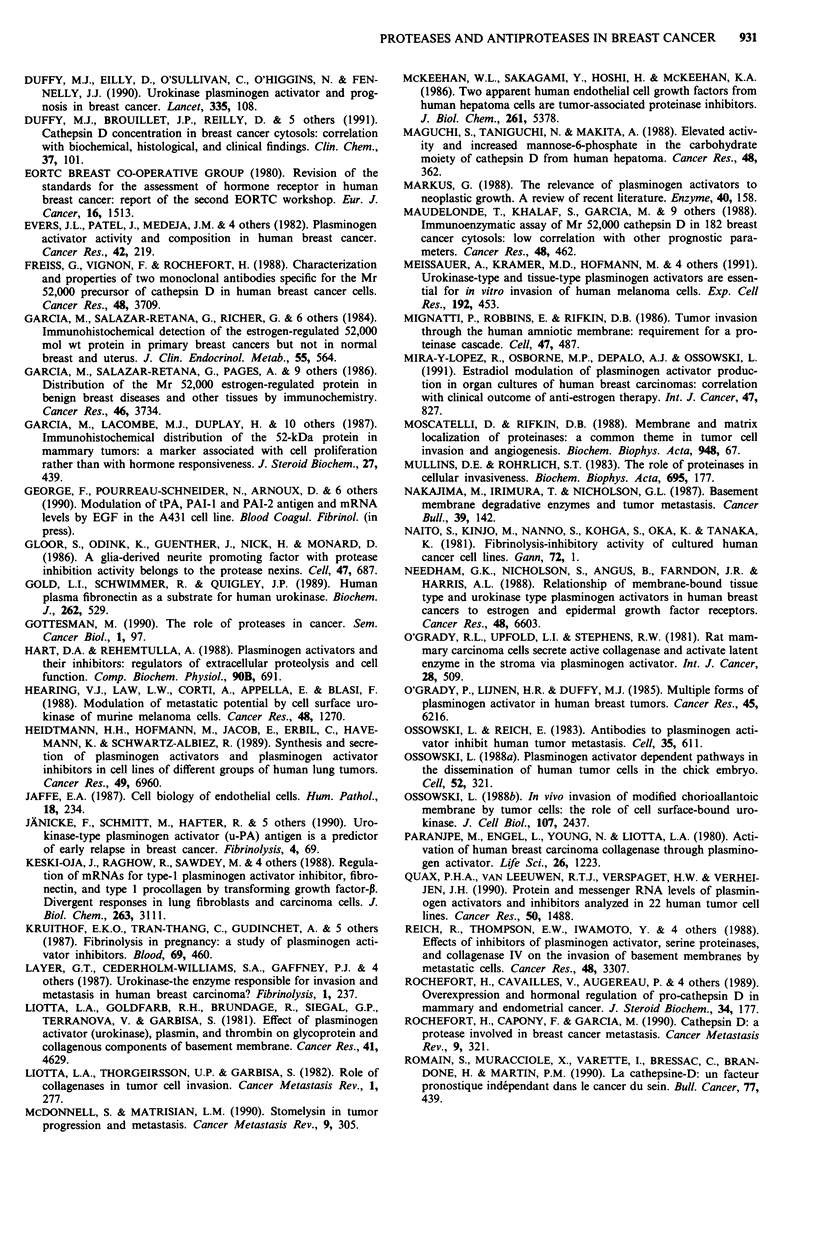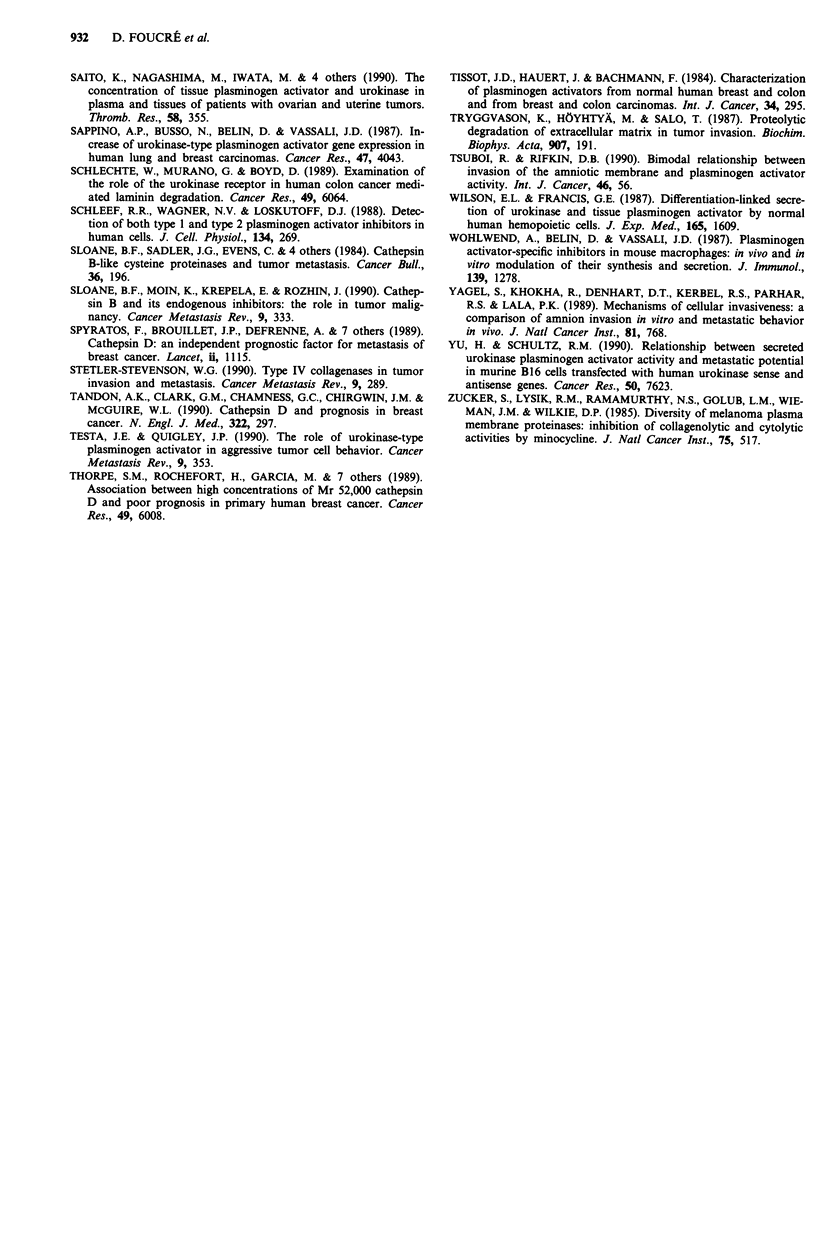# Relationship between cathepsin D, urokinase, and plasminogen activator inhibitors in malignant vs benign breast tumours.

**DOI:** 10.1038/bjc.1991.428

**Published:** 1991-11

**Authors:** D. Foucré, C. Bouchet, K. Hacène, N. Pourreau-Schneider, A. Gentile, P. M. Martin, A. Desplaces, J. Oglobine

**Affiliations:** Laboratoire d'immunochimie, Centre René Huguenin, Saint-Cloud, France.

## Abstract

The concentrations of cathepsin D (Cath D), urokinase (uPA) and two plasminogen activator inhibitors (PAI-1 and PAI-2) were analysed in the cytosols of 130 human mammary tumours (43 benign tumours and 87 primary and unilateral breast carcinomas). uPA, PAI-1 and PAI-2 levels were measured by antigenic immunoassays and Cath D by immunoradiometric assay. The median levels of the four parameters were significantly higher in the malignant tumours than in the benign ones. Cath D and uPA increases were 4-fold and 5-fold respectively. PAI-1 and PAI-2 increases were much more important, 74-fold and 29-fold respectively. In malignant tumours, median levels of Cath D and uPA did not vary according to classical prognostic factors (histologic grade, presence or absence of axillary lymph nodes, steroid receptors, UICC stage, tumour size, age, and menopausal status). However, PAI-1 decreased in ER+ and PR+ tumours and PAI-2 increased in menopausal women's tumours. When Cath D, uPA, PAI-1 and PAI-2 levels in malignant tumours were compared, positive correlations were found for all combinations. The implication of plasminogen activator inhibitors in the phenomenon was surprising and merits further investigation using tools other than global antigen measurements in tumours.


*Br. J. Cancer* (1991), **64**, 926–932 © Macmillan Press Ltd., 1991

### Relationship between cathepsin D, urokinase, and plasminogen activator inhibitors in malignant *vs* benign breast tumours

D. Foucré[1], C. Bouchet[1], K. Hacène[2], N. Pourreau-Schneider[4], A. Gentile[3], P.M. Martin[4], A. Desplaces[1] & J. Oglobine[1]

[1]*Laboratoire d'immunochimie,* [2]*Unité de statistique,* [3]*Laboratoire d'anatomo-pathologie, Centre René Huguenin, 35, rue Dailly, 92211 Saint-Cloud;* [4]*Laboratorie de Cancérologie Expérimentale, Faculté de Médecine Nord, Boulevard P. Dramard, 13326 Marseille, France.*

**Summary** The concentrations of cathepsin D (Cath D), urokinase (uPA) and two plasminogen activator inhibitors (PAI-1 and PAI-2) were analysed in the cytosols of 130 human mammary tumours (43 benign tumours and 87 primary and unilateral breast carcinomas). uPA, PAI-1 and PAI-2 levels were measured by antigenic immunoassays and Cath D by immunoradiometric assay. The median levels of the four parameters were significantly higher in the malignant tumours than in the benign ones. Cath D and uPA increases were 4-fold and 5-fold respectively. PAI-1 and PAI-2 increases were much more important, 74-fold and 29-fold respectively. In malignant tumours, median levels of Cath D and uPA did not vary according to classical prognostic factors (histologic grade, presence or absence of axillary lymph nodes, steroid receptors, UICC stage, tumour size, age, and menopausal status). However, PAI-1 decreased in $ER^+$ and $PR^+$ tumours and PAI-2 increased in menopausal women's tumours. When Cath D, uPA, PAI-1 and PAI-2 levels in malignant tumours were compared, positive correlations were found for all combinations. The implication of plasminogen activator inhibitors in the phenomenon was surprising and merits further investigation using tools other than global antigen measurements in tumours.

Proteases play a role in metastatic dissemination. They contribute to basement membrane and connective tissue degradation, allowing vascular endothelial crossover (for review: Mullins & Rohrlich, 1983; Tryggvason *et al.*, 1987; Moscatelli & Rifkin, 1988; Gottesman, 1990). Diverse enzymes are produced in abundance by malignant cells and are implicated in tumour cell invasion: collagenases (for review: Liotta *et al.*, 1982; Stetler-Stevenson, 1990); stromelysin (McDonnel & Matrisian, 1990); cathepsin B (Sloane *et al.*, 1984; 1990); cathepsin D (Maguchi *et al.*, 1988; Rochefort *et al.*, 1990); heparanase (Nakajima *et al.*, 1987); urokinase type plasminogen activator (for review: Dano *et al.*, 1985; Markus, 1988; Testa & Quigley, 1990). Their proteolytic activities are often concentrated in the pericellular environment or cell surface bound (Zucker *et al.*, 1985).

Among these enzymes, urokinase (uPA) has been extensively studied. uPA is a serine protease that transforms plasminogen into plasmin which is active on a large number of substrates. It can degrade basement membrane components (Liotta *et al.*, 1981) and can activate type IV procollagenase (Paranjpe *et al.*, 1980; O'Grady *et al.*, 1981). uPA can by itself degrade fibronectin (Gold *et al.*, 1989). Inhibition of uPA using anti-urokinase antibodies has shown the importance of uPA in tumour invasion (Ossowski & Reich, 1983). The inhibition of uPA activity prevents laminin degradation (Boyd *et al.*, 1989) and invasion of amniotic membrane (Mignatti *et al.*, 1986; Yagel *et al.*, 1989; Tsuboi & Rifkin, 1990) and of extracellular matrix (Meissauer *et al.*, 1991). Moreover, transfection of uPA gene into H-*ras*-transformed fibroblasts, mouse L cells, and murine melanoma cells enhances invasion and metastasis of these cells (Axelrod *et al.*, 1989; Cajot *et al.*, 1989; Yu & Schultz, 1990). During the metastatic process, uPA is especially potent in mesenchymal infiltration and intravasation by tumour cells (Ossowski, 1988*a*). uPA bound to membrane receptors is more active than free uPA in matrix degradation (Ossowski, 1988*b*; Hearing *et al.*, 1988; Schlechte *et al.*, 1989).

uPA activity is controlled by several specific inhibitors, PAI-1, PAI-2, PAI-3, protease nexin (for review: Hart & Rehemtulla, 1988). There are few studies on these inhibitors in malignant tissues, even though they have been detected in variable amounts in several cancer cell lines: bladder, lung, kidney, stomach (Naito *et al.*, 1981); breast, uterus, lymphoma, epidermoid carcinoma (Cajot *et al.*, 1986); neuroblastoma (Benjamin *et al.*, 1989). Other cancer cell lines are completely devoid of plasminogen activator inhibitors (Quax *et al.*, 1990). In two mammary carcinoma cell lines, Cajot *et al.* (1986) found PAI in T47D cells but not in MCF7 cells.

Cathepsin D (Cath D) is an aspartyl protease for which the normal function is protein degradation in lysosomes. In malignant cells, Cath D is also secreted (Maguchi *et al.*, 1988; Capony *et al.*, 1989; Rochefort *et al.*, 1989). Cath D in conditioned media from certain cell lines can degrade extracellular matrix (Briozzo *et al.*, 1988).

uPA and Cath D have been independently implicated in malignant progression, but until a recent report by Duffy *et al.* (1991), no studies have been made on detection of both proteases in the same tumours. In addition, since little is known of PAIs in tumours, we assayed in 43 benign and 87 malignant tumours, the concomitant production of uPA, Cath D, PAI-1 and PAI-2. In the malignant population, they were studied as a function of clinical, histological and biochemical factors.

### Patients and methods

### Patients

One hundred and thirty patients with primary and unilateral breast tumours were selected before treatment (43 benign tumours and 87 malignant tumours). For every patient, age, UICC stage, Scarff, Bloom and Richardson (1957) histological grade, number of involved lymph nodes, tumour size, menopausal status, oestrogen and progesterone receptor status were determined.

### Tissue extraction

Tumours were snap frozen in liquid nitrogen after surgery (tumourectomy or mastectomy). Tissues were pulverised at

Correspondence: D. Fourcré, Centre R. Huguenin, 35, rue Dailly, 92211 Saint Cloud, France.
Received 29 January 1991; and in revised form 4 July 1991.

4°C in 50 mM Tris pH 7.4, Triton X-100 (0.1%), Tween 80 (0.01%), 0.05 μM aprotinin buffer (10 ml $g^{-1}$ tumour). The homogenate was ultracentrifuged (105,000 *g* for 1 h). They cytosols were collected and stored at −80°C.

### *Assay of Cath D*

Cath D was determined in breast tumour cytosols using an immunoradiometric assay (ELSA-Cath D, CIS Bioindustries, Gif-sur-Yvette, France). The first monoclonal antibody was coated in the solid phase and the second monoclonal antibody, raised against two different sites of Cath D heavy chain, was radiolabelled with 125 iodine. This assay measured pro-cathepsin D (52 kD), mature Cath D (48 kD) and Cath D heavy chain (34 kD). Cytosols were diluted 1/100 and incubated with the two monoclonal antibodies at 37°C with agitation for 2 h. After three washes, the radioactivity was measured with a gamma scintillation counter. Results were obtained from a standard curve under the same conditions. Assays were performed in duplicate.

### *Assay of uPA, PAI-1 and PAI-2*

Antigens were measured by commercially available ELISA kits (TINT ELISE Biopool, Umea, Sweden). Microtiter plates were coated with monoclonal antibodies raised against:
- For uPA assay: pro-urokinase, 33 kD uPA, 50 kD uPA, and uPA bound to PAI.
- For PAI-1 assay: active and latent PAI-1, and PAI-1 bound to uPA and tPA.
- For PAI-2 assay: non glycosylated (47 kD) and glycosylated (60 kD) forms.

Cytosols, diluted 1/2 for uPA and PAI-2 antigens and 1/6 for PAI-1, were incubated for 3 h at room temperature with agitation. Then, the second polyclonal antibody labelled with peroxidase was added for 1 h with agitation. After three washes, antigens were revealed with ortho-phenylenediamine. The reaction was stopped with sulphuric acid. Results were obtained from standard curves under the same conditions. Assays were performed in triplicate.

### *Oestrogen and progesterone receptor assays*

Steroid receptors were measured by the dextran-coated charcoal method recommended by EORTC (1980). A cut-off level of 10 fmol $mg^{-1}$ protein was used to determine positive or negative receptor status.

### *Protein determination*

Protein levels were assayed using the Bradford method (Bradford, 1976, Bio-Rad, California, USA).

### *Statistical analysis*

The analysis of differences was performed using Student's *t*-test. Correlations were calculated by Spearman's method (correlation coefficient rs) and Pearson's method (correlation coefficient rp). All tests were performed at a significance level of $P = 0.05$.

### Results

### *Cath D, uPA, PAI-1, and PAI-2 concentrations in benign and malignant breast tumours (Table I)*

*Cath D (Figure 1) and uPA (Figure 2)* All of the benign and malignant tumours contained Cath D. uPA was present in all malignant tumours and in 86% of benign tumours.

The mean levels of Cath D and uPA were significantly higher in malignant tumours than in benign tumours ($P < 0.00001$). The increase was approximately the same for the two proteases: about 4-fold for Cath D and 5-fold for uPA.

*PAI-1 (Figure 3) and PAI-2 (Figure 4)* 71% of benign tumours had neither PAI-1 nor PAI-2. Twenty-seven per cent of benign tumours contained only PAI-1 or PAI-2. PAI-1 was present in 10% of benign tumours and PAI-2 in 22%. A single benign tumour produced both inhibitors.

Seven per cent of malignant tumours had neither PAI-1 nor PAI-2. PAI-1 was present in 80% of malignant tumours and PAI-2 in 70%. Fifty-seven per cent contained both inhibitors. Thirty-seven per cent contained only one of the two inhibitors.

Mean concentrations of PAI-1 and PAI-2 were significant-

**Figure 1** Cathepsin D content in cytosols of mammary tumours. Horizontal bars indicate median values.

**Table I** Mean concentrations of Cath D, uPA, PAI-1 and PAI-2 in benign and malignant breast tumours

| *Tumours* | *Cath D*[a] | *Standard deviation* | *UPA*[b] | *Standard deviation* | *PAI-1*[b] | *Standard deviation* | *PAI-2*[b] | *Standard deviation* |
|---|---|---|---|---|---|---|---|---|
| Benign $n = 43$ | 16.58 | 17.07 | 0.29 | 0.26 | 0.02 | 0.06 | 0.17 | 0.42 |
| Malignant $n = 87$ | 70.68 | 42.26 | 1.52 | 1.23 | 1.48 | 2.33 | 5 | 11.73 |
| | $P = 0.00001$ | | $P = 0.00001$ | | $P = 0.00015$ | | $P = 0.0087$ | |

[a]Mean concentration pmol $mg^{-1}$ protein. [b]Mean concentration ng $mg^{-1}$ protein.

**Figure 2** uPA content in cytosols of mammary tumours. Horizontal bars indicate median values.

ly higher in malignant than in benign tumours (respectively $P = 0.00015$ and $P = 0.0087$). These increases were elevated: about 74-fold for PAI-1 and 29-fold for PAI-2.

*Relation between Cath D, uPA, PAI-1, PAI-2 and histological, clinical and biochemical parameters in malignant tumours (Table II)*

*Cath D and uPA* The mean concentration of Cath D and uPA were independent of patient's age and menopausal status, tumour stage, grading and size, lymph node involvement, oestrogen and progesterone receptor status.

*PAI-1 and PAI-2* PAI-1 varied only with steroid receptor status, and was independent of other classical prognostic factors. The mean concentration of PAI-1 was significantly lower when the tumours lacked oestrogen ($P = 0.001$) and progesterone ($P = 0.016$) receptors.

PAI-2 varied only according to patient's hormonal status. Menopausal women's tumours contained 4-fold more PAI-2 than non-menopausal women's tumours ($P = 0.036$).

*Correlation between Cath D, uPA, PAI-1, PAI-2 in benign and malignant tumours*

In benign tumours, Cath D and uPA concentrations were significantly and linearly correlated (rs = 0.583, $P = 0.00011$; rp = 0.486, $P = 0.0016$). Due to the absence of PAI-1 and PAI-2 in the majority of benign tumours, no correlation could be found.

In malignant tumours, Cath D and uPA concentrations were significantly, but nonlinearly correlated (Table III). They were also significantly correlated with concentrations of PAI-1 and PAI-2. Cath D was strongly linearly correlated with PAI-1 and nonlinearly correlated with PAI-2. uPA was nonlinearly correlated with PAI-1 and PAI-2. There was a clear linear correlation between PAI-1 and PAI-2.

**Table II** Levels of Cath D, uPA, PAI-1 and PAI-2 compared with histological, clinical, and biochemical factors in malignant tumours

| *Factors* | *Number of patients* | *Cath D*[a] | *Standard deviation* | *UPA*[b] | *Standard deviation* | *PAI-1*[b] | *Standard deviation* | *PAI-2*[b] | *Standard deviation* |
|---|---|---|---|---|---|---|---|---|---|
| Age ($n = 87$) | | | | | | | | | |
| < 50 years | 29 | 66.46 | 36.85 | 1.32 | 0.80 | 1.69 | 2.85 | 1.55 | 2.79 |
| 50–65 years · | 26 | 68.41 | 43.05 | 1.92 | 1.93 | 1.29 | 1.66 | 4.20 | 9.86 |
| > 65 years | 32 | 76.41 | 46.70 | 1.37 | 0.66 | 1.43 | 2.35 | 8.63 | 16.32 |
| UICC stage ($n = 87$) | | | | | | | | | |
| I | 17 | 54.62 | 30.17 | 1.24 | 0.85 | 0.54 | 0.68 | 1.46 | 2.57 |
| II | 62 | 75.23 | 43.54 | 1.58 | 1.37 | 1.73 | 2.66 | 6.38 | 13.67 |
| III | 8 | 70.72 | 50.76 | 1.64 | 0.65 | 1.51 | 1.25 | 2.10 | 2.44 |
| Scarff, Bloom & Richardson grading ($n = 82$) | | | | | | | | | |
| I | 14 | 52.97 | 36.61 | 1.41 | 0.98 | 0.67 | 0.86 | 4.21 | 5.05 |
| II | 42 | 72.3 | 39.14 | 1.47 | 0.91 | 1.48 | 2.88 | 6.04 | 14.86 |
| III | 26 | 84.88 | 46.45 | 1.83 | 1.75 | 2.16 | 1.89 | 4.59 | 9.69 |
| Number of lymph nodes involved ($n = 85$) | | | | | | | | | |
| 0 | 38 | 68.6 | 40.91 | 1.35 | 0.90 | 1.58 | 3.17 | 6.96 | 15.63 |
| 1–3 | 26 | 65.45 | 38.10 | 1.88 | 1.86 | 1.23 | 1.07 | 3.48 | 6.13 |
| > 3 | 21 | 87.49 | 47.04 | 1.48 | 0.59 | 1.72 | 1.77 | 3.82 | 8.94 |
| Tumour size ($n = 87$) | | | | | | | | | |
| < 20 mm | 22 | 55.06 | 34.75 | 1.67 | 1.00 | 1.02 | 1.51 | 4.17 | 6.38 |
| 20–37 mm | 51 | 73.76 | 40.20 | 1.51 | 1.43 | 1.60 | 2.78 | 6.25 | 14.72 |
| > 37 mm | 14 | 83.13 | 54.75 | 1.31 | 0.71 | 1.73 | 1.51 | 1.91 | 2.68 |
| Menopausal status ($n = 87$) | | | | | | | | | |
| premenopausal | 36 | 68.54 | 37.00 | 1.33 | 0.77 | 1.63 | 2.64 | 1.85[e] | 3.18 |
| postmenopausal | 51 | 72.19 | 45.91 | 1.65 | 1.46 | 1.37 | 2.12 | 7.20 | 14.73 |
| Oestrogen receptors ($n = 84$) | | | | | | | | | |
| ER + | 60 | 69.44 | 42.68 | 1.37 | 0.87 | 0.96[c] | 1.14 | 4.10 | 7.39 |
| ER − | 24 | 77.67 | 41.26 | 1.90 | 1.85 | 2.79 | 3.80 | 7.94 | 19.60 |
| Progesterone receptors ($n = 84$) | | | | | | | | | |
| PR + | 62 | 69.63 | 43.52 | 1.39 | 0.90 | 1.12[d] | 1.37 | 4.24 | 8.06 |
| PR − | 22 | 78.31 | 38.11 | 1.90 | 1.89 | 2.52 | 3.91 | 7.71 | 19.28 |

[a]Mean level pmol $mg^{-1}$ protein; [b]Mean level ng $mg^{-1}$ protein; [c]Significant difference $P = 0.001$; [d]Significant difference $P = 0.016$; [e]Significant difference $P = 0.036$.

**Figure 3** PAI-1 content in cytosols of mammary tumours. Horizontal bars indicate median values.

**Figure 4** PAI-2 content in cytosols of mammary tumours. Horizontal bars indicate median values.

### Discussion

One originality in this study was the concomitant measurement of uPA, Cath D, PAI-1 and PAI-2 in a series of benign and malignant tumours. The major findings were the significant increases of the two proteases and particularly of two anti-proteases in malignant compared to benign breast tumours and the positive correlation for all combinations.

Our results are in agreement with those of others concerning the two enzymes. Individually uPA and Cath D levels have been found to be elevated in malignant tumours compared to benign counterparts. Urokinase concentration increase (activity, antigen, mRNA) in malignant breast tumours was observed by many authors (Evers *et al.*, 1982; O'Grady *et al.*, 1985; Layer *et al.*, 1987; Sappino *et al.*, 1987; Jänicke *et al.*, 1990). Cath D concentration was also higher in malignant breast tumours compared to benign ones (Abecassis *et al.*, 1984; Duffy *et al.*, 1991) and to normal mammary tissue (Capony *et al.*, 1989; Tandon *et al.*, 1990; Duffy *et al.*, 1991). In addition, Duffy *et al.* (1991) recently found a simultaneous rise of uPA and Cath D concentrations in a series of malignant breast tumours. This positive correlation that we also found in malignant tumours suggests the possible intervention of uPA and Cath D in tumour invasion. Mignatti *et al.* (1986) and Reich *et al.* (1988) have previously shown the existence of a proteolytic cascade (plasminogen activators, plasmin, type IV collagenase) for basal membrane degradation *in vitro*.

High levels of cytosolic uPA (Duffy *et al.*, 1990; Jänicke *et al.*, 1990) and Cath D (Spyratos *et al.*, 1989; Tandon *et al.*, 1989; Thorpe *et al.*, 1989; Romain *et al.*, 1990; Duffy *et al.*, 1991) in breast cancers have been associated with shorter disease-free and overall survival. We, therefore, looked for relationships between proteases and anti-proteases as a function of classical prognostic factors. Like others, we found no significant variation of uPA (O'Grady *et al.*, 1985; Duffy *et al.*, 1986; Sappino *et al.*, 1987; Needham *et al.*, 1988; Duffy *et al.*, 1990; Jänicke *et al.*, 1990; Mira-y-Lopez *et al.*, 1991) or Cath D (Abecassis *et al.*, 1984; Maudelonde *et al.*, 1988; Brouillet *et al.*, 1990; Romain *et al.*, 1990) concentrations with tumour grade, lymph node invasion, tumour size or steroid hormone receptors. In contrast, we found that PAI-1 concentration decreased in tumours with oestrogen or progesterone receptors, whereas PAI-2 increased significantly in malignant tumours of post-menopausal patients compared with non-menopausal patients. Hormonal regulation of PAIs has already been reported (for review: Adreasen *et al.*, 1990). However, Cohen *et al.* (1989) have found that PAI-1 and PAI-2 are independently regulated. The hormonal regulation of PAIs could possibly explain the correlation that we found between Cath D and PAI increase in malignant tumours, because Rochefort *et al.* (1989) have demonstrated the induction of Cath D by oestrogen.

Jänicke *et al.* (1990) have also reported a significant increase of PAI-1 concentration in malignant breast tumours. However their results differ from ours in that they found no correlation between urokinase and PAI-1 concentrations. In addition, the increase that they found for PAI-1 was small (about 10-fold) compared to the 74-fold increase that we found in malignant over benign tumours. This discrepancy may be due to different buffers and anti-urokinase antibodies used.

The very high PAI-1 and PAI-2 increases that occurred with malignancy may counteract urokinase-mediated tissue degradation by tumour cells. *In vitro* studies have shown that PAI-1 and PAI-2 can inhibit plasminogen-dependent extracellular matrix degradation by colon carcinoma cells (Cajot *et al.*, 1990; Baker *et al.*, 1990). However, the function of these inhibitors in malignancy is puzzling. Maybe they have an activity other than that of inhibiting PAs. GdNPF (glia-derived neurite promoting factor) which inhibits PAs, also regulates the migration of neuronal cells (Gloor *et al.*, 1986). Other small molecular weight serine protease inhibitors, secreted by human hepatoma cells, stimulated endothelial cell growth (McKeehan *et al.*, 1986). If such functions should

**Table III** Correlations between concentrations of Cath D, uPA, PAI-1 and PAI-2 in malignant tumours

| *Factors* | *uPA* | *PAI-1* | *PAI-2* |
|---|---|---|---|
| Cath D | rs = 0.298 $P<0.003$<br>rp = 0.145 $P = 0.15$ | rs = 0.473 $P<0.0001$<br>rp = 0.245 $P = 0.015$ | rs = 0.256 $P = 0.011$<br>rp = 0.119 $P = 0.25$ |
| uPA | | rs = 0.282 $P = 0.004$<br>rp = 0.158 $P = 0.11$ | rs = 0.270 $P = 0.007$<br>rp = 0.03 $P = 0.77$ |
| PAI-1 | | | rs = 0.233 $P = 0.026$<br>rp = 0.469 $P<0.0001$ |

rs: Spearman correlation coefficient; rp: Pearson correlation coefficient. $P>0.05$ non significant.

exist for PAI, they could promote tumour cell invasiveness.

Since our technique measured global enzyme and inhibitor levels in cytosols of pulverised tumours, we can ask the question about the tissue compartment responsible for their production. uPA, Cath D and PAIs have been found in plasma (Saito *et al.*, 1990; Freiss *et al.*, 1988; Kruithof *et al.*, 1987), and in many types of normal cells (Bernik *et al.*, 1981; Jaffe, 1987; Chapman *et al.*, 1982; Bergman *et al.*, 1986; Keski-Oja *et al.*, 1988; Wilson *et al.*, 1987; Tissot *et al.*, 1984; Wohlwend *et al.*, 1987). However, we can consider that the tumour cells are the major producer. Saito *et al.* (1990) have shown that the plasmatic concentration of uPA did not vary between patients with ovarian or uterine benign and malignant tumours. By immunohistochemistry, Jänicke *et al.* (1990) and Costantini *et al.* (1991) have found uPA in the cytoplasma and the plasma membrane of breast tumour cells. For Cath D an immunohistochemistry study of benign and malignant breast tumours has shown that procathepsin D staining was more intense in malignant cells than in benign mastopathies (Garcia *et al.*, 1987). The distribution of Cath D in human breast appears to be relatively specific for mammary epithelial cells and to be associated with tumour development (Garcia *et al.*, 1984; Garcia *et al.*, 1986). Besides, in culture, uPA, Cath D, and PAI-1 are produced by many breast carcinoma cell lines (Tissot *et al.*, 1984; Cajot *et al.*, 1986; Quax *et al.*, 1990; Briozzo *et al.*, 1988). Moreover, Costantini *et al.* (1991) have observed PAI-1 in breast tumour cells using immunohistochemical technique. To our knowledge PAI-2 has not been reported in breast carcinoma cell but it is produced by other carcinoma cell lines (Schleef *et al.*, 1988; Heidtmann *et al.*, 1989; George *et al.*, 1990).

Further studies on the concomitant production of proteases and anti-proteases in malignant tumours should shed a light on their role in matrix degradation.

Thanks are due to Dr M. Pagano for his critical reading of the manuscript and to Mrs F. Spyratos for her assistance. We also thank Pr J. Rouëssé, Director of centre R. Huguenin and Dr J. Gest, President of the Association pour la Recherche contre le Cancer de Saint-Cloud for their support. This work was supported by the Ligue Nationale de Lutte contre le Cancer (Comité des Hauts de Seine).

### References

ABECASSIS, J., COLLARD, R., EBER, M., PUSEL, J., FRICKER, J.P. & METHLIN, G. (1984). Proteinases and sialyltransferase in human breast tumors. *Int. J. Cancer*, **33**, 821.

ANDREASEN, P.A., GEORG, B., LUND, L.R., RICCIO, A. & STACEY, S.N. (1990). Plasminogen activator inhibitors: hormonally regulated serpins. *Mol. Cell Endocrinol.*, **68**, 1.

AXELROD, J.H., REICH, R. & MISKIN, R. (1989). Expression of human recombinant plasminogen activators enhances invasion and experimental metastasis of H-*ras*-transformed NIH3T3 cells. *Mol. Cell Biol.*, **9**, 2133.

BAKER, M.S., BLEAKLEY, P., WOODROW, G.C. & DOE, W.F. (1990). Inhibition of cancer cell urokinase plasminogen activator by its specific inhibitor PAI-2 and subsequent effects on extracellular matrix degradation. *Cancer Res.*, **50**, 4676.

BENJAMIN, L.A., McGARRY, R.C. & HART, D.A. (1989). Effect of retinoic acid on human neuroblastoma: correlation between morphological differentiation and changes in plasminogen activator and inhibitor activity. *Cancer Chemother. Pharmacol.*, **25**, 25.

BERGMAN, B.L., SCOTT, R.W., BAJPAI, A., WATTS, S. & BAKER, J.B. (1986). Inhibition of tumor-cell-mediated extracellular matrix destruction by a fibroblast proteinase inhibition, protease nexin 1. *Proc. Natl Acad. Sci. USA*, **83**, 996.

BERNIK, M.B., WIJNGAARDS, G. & RIJKEN, D.C. (1981). Production by human tissues in cultures of immunologically distinct, multiple molecular weight forms of plasminogen activators. *Ann. NY Acad. Sci.*, **370**, 592.

BLOOM, H.J.G. & RICHARDSON, W.W. (1957). Histological grading and prognosis in breast cancer. A study of 1409 cases of which 359 have been followed for 15 years. *Br. J. Cancer*, **11**, 35.

BOYD, D., ZIOBER, B., CHAKRABARTY, S. & BRATTAIN, M. (1989). Examination of urokinase protein/transcript levels and their relationship with laminin degradation in cultured colon carcinoma. *Cancer Res.*, **49**, 816.

BRADFORD, M.M. (1976). A rapid and sensitive method for the quantitation of microgram quantities of protein utilizing the principle of protein-dye binding. *Annal. Biochem.*, **72**, 248.

BRIOZZO, P., MORISSET, M., CAPONY, F., ROUGEOT, C. & ROCHEFORT, H. (1988). *In vitro* degradation of extracellular matrix with Mr 52,000 cathepsin D secreted by breast cancer cells. *Cancer Res.*, **48**, 3688.

BROUILLET, J.P., THEILLET, C., MAUDELONDE, T. & 6 others (1990). Cathepsin D assay in primary breast cancer and lymph nodes: relationship with c-*myc*, c-*erb*-B-2 and *int*-2 oncogene amplification and node invasiveness. *Eur. J. Cancer*, **26**, 437.

CAJOT, J.F., KRUITHOF, E.K.O., SCHLEUNING, W.D., SORDAT, B. & BACHMANN, F. (1986). Plasminogen activators, plasminogen activator inhibitors and procoagulant analyzed in twenty human tumor cell lines. *Int. J. Cancer*, **38**, 719.

CAJOT, J.F., SCHLEUNING, W.D., MEDCALF, R.F. & 4 others (1989). Mouse L cells expressing human prourokinase-type plasminogen activator: effects on extracellular matrix degradation and invasion. *J. Cell Biol.*, **109**, 915.

CAJOT, J.F., BAMAT, J., BERGONZELLI, G.E. & 4 others (1990). Plasminogen-activator inhibitor type 1 is a potent natural inhibitor of extracellular matrix degradation by fibrosarcoma and colon carcinoma cells. *Proc. Natl Acad. Sci. USA*, **87**, 6939.

CAPONY, F., ROUGEOT, C., MONTCOURRIER, P., CAVAILLES, V., SALAZAR, G. & ROCHEFORT, H. (1989). Increased secretion, altered processing, and glycosylation of pro-cathepsin D in human mammary cancer cells. *Cancer Res.*, **49**, 3904.

CHAPMAN, H.A., VAVRIN, Z. & HIBBS, J.B. (1982). Macrophage fibrinolytic activity: identification of two pathways of plasmin formation by intact cells and of a plasminogen activator inhibitor. *Cell*, **28**, 653.

COHEN, R.L., NICLAS, J., LEE, W.M.F. & 5 others (1989). Effect of transformation on expression of plasminogen activator inhibitors 1 and 2: evidence for independent regulation. *J. Biol. Chem.*, **264**, 8375.

COSTANTINI, V., ZACHARSKI, L.R., MEMOLI, V.A., KUDRYK, B.J., ROUSSEAU, S.M. & STUMP, D. (1991). Occurrence of components of fibrinolysis pathways *in situ* in neoplastic and non neoplastic human breast tissue. *Cancer Res.*, **51**, 354.

DANO, K., ANDREASEN, P.,A., GRONDAHL-HANSEN, J., KRISTENSEN, P., NIELSEN, L.S. & SKRIVER, L. (1985). Plasminogen activators, tissue degradation, and cancer. *Adv. Cancer Res.*, **44**, 139.

DUFFY, M.J., O'GRADY, P., SIMON, J., ROSE, M. & LIJNEN, H.R. (1986). Tissue-type plasminogen activator in breast cancer: relationship with estradiol and progesterone receptors. *J. Natl Cancer Inst.*, **77**, 621.

DUFFY, M.J., EILLY, D., O'SULLIVAN, C., O'HIGGINS, N. & FENNELLY, J.J. (1990). Urokinase plasminogen activator and prognosis in breast cancer. *Lancet*, **335**, 108.

DUFFY, M.J., BROUILLET, J.P., REILLY, D. & 5 others (1991). Cathepsin D concentration in breast cancer cytosols: correlation with biochemical, histological, and clinical findings. *Clin. Chem.*, **37**, 101.

EORTC BREAST CO-OPERATIVE GROUP (1980). Revision of the standards for the assessment of hormone receptor in human breast cancer: report of the second EORTC workshop. *Eur. J. Cancer*, **16**, 1513.

EVERS, J.L., PATEL, J., MEDEJA, J.M. & 4 others (1982). Plasminogen activator activity and composition in human breast cancer. *Cancer Res.*, **42**, 219.

FREISS, G., VIGNON, F. & ROCHEFORT, H. (1988). Characterization and properties of two monoclonal antibodies specific for the Mr 52,000 precursor of cathepsin D in human breast cancer cells. *Cancer Res.*, **48**, 3709.

GARCIA, M., SALAZAR-RETANA, G., RICHER, G. & 6 others (1984). Immunohistochemical detection of the estrogen-regulated 52,000 mol wt protein in primary breast cancers but not in normal breast and uterus. *J. Clin. Endocrinol. Metab.*, **55**, 564.

GARCIA, M., SALAZAR-RETANA, G., PAGES, A. & 9 others (1986). Distribution of the Mr 52,000 estrogen-regulated protein in benign breast diseases and other tissues by immunochemistry. *Cancer Res.*, **46**, 3734.

GARCIA, M., LACOMBE, M.J., DUPLAY, H. & 10 others (1987). Immunohistochemical distribution of the 52-kDa protein in mammary tumors: a marker associated with cell proliferation rather than with hormone responsiveness. *J. Steroid Biochem.*, **27**, 439.

GEORGE, F., POURREAU-SCHNEIDER, N., ARNOUX, D. & 6 others (1990). Modulation of tPA, PAI-1 and PAI-2 antigen and mRNA levels by EGF in the A431 cell line. *Blood Coagul. Fibrinol.* (in press).

GLOOR, S., ODINK, K., GUENTHER, J., NICK, H. & MONARD, D. (1986). A glia-derived neurite promoting factor with protease inhibition activity belongs to the protease nexins. *Cell*, **47**, 687.

GOLD, L.I., SCHWIMMER, R. & QUIGLEY, J.P. (1989). Human plasma fibronectin as a substrate for human urokinase. *Biochem. J.*, **262**, 529.

GOTTESMAN, M. (1990). The role of proteases in cancer. *Sem. Cancer Biol.*, **1**, 97.

HART, D.A. & REHEMTULLA, A. (1988). Plasminogen activators and their inhibitors: regulators of extracellular proteolysis and cell function. *Comp. Biochem. Physiol.*, **90B**, 691.

HEARING, V.J., LAW, L.W., CORTI, A., APPELLA, E. & BLASI, F. (1988). Modulation of metastatic potential by cell surface urokinase of murine melanoma cells. *Cancer Res.*, **48**, 1270.

HEIDTMANN, H.H., HOFMANN, M., JACOB, E., ERBIL, C., HAVEMANN, K. & SCHWARTZ-ALBIEZ, R. (1989). Synthesis and secretion of plasminogen activators and plasminogen activator inhibitors in cell lines of different groups of human lung tumors. *Cancer Res.*, **49**, 6960.

JAFFE, E.A. (1987). Cell biology of endothelial cells. *Hum. Pathol.*, **18**, 234.

JÄNICKE, F., SCHMITT, M., HAFTER, R. & 5 others (1990). Urokinase-type plasminogen activator (u-PA) antigen is a predictor of early relapse in breast cancer. *Fibrinolysis*, **4**, 69.

KESKI-OJA, J., RAGHOW, R., SAWDEY, M. & 4 others (1988). Regulation of mRNAs for type-1 plasminogen activator inhibitor, fibronectin, and type 1 procollagen by transforming growth factor-β. Divergent responses in lung fibroblasts and carcinoma cells. *J. Biol. Chem.*, **263**, 3111.

KRUITHOF, E.K.O., TRAN-THANG, C., GUDINCHET, A. & 5 others (1987). Fibrinolysis in pregnancy: a study of plasminogen activator inhibitors. *Blood*, **69**, 460.

LAYER, G.T., CEDERHOLM-WILLIAMS, S.A., GAFFNEY, P.J. & 4 others (1987). Urokinase-the enzyme responsible for invasion and metastasis in human breast carcinoma? *Fibrinolysis*, **1**, 237.

LIOTTA, L.A., GOLDFARB, R.H., BRUNDAGE, R., SIEGAL, G.P., TERRANOVA, V. & GARBISA, S. (1981). Effect of plasminogen activator (urokinase), plasmin, and thrombin on glycoprotein and collagenous components of basement membrane. *Cancer Res.*, **41**, 4629.

LIOTTA, L.A., THORGEIRSSON, U.P. & GARBISA, S. (1982). Role of collagenases in tumor cell invasion. *Cancer Metastasis Rev.*, **1**, 277.

McDONNELL, S. & MATRISIAN, L.M. (1990). Stomelysin in tumor progression and metastasis. *Cancer Metastasis Rev.*, **9**, 305.

McKEEHAN, W.L., SAKAGAMI, Y., HOSHI, H. & McKEEHAN, K.A. (1986). Two apparent human endothelial cell growth factors from human hepatoma cells are tumor-associated proteinase inhibitors. *J. Biol. Chem.*, **261**, 5378.

MAGUCHI, S., TANIGUCHI, N. & MAKITA, A. (1988). Elevated activity and increased mannose-6-phosphate in the carbohydrate moiety of cathepsin D from human hepatoma. *Cancer Res.*, **48**, 362.

MARKUS, G. (1988). The relevance of plasminogen activators to neoplastic growth. A review of recent literature. *Enzyme*, **40**, 158.

MAUDELONDE, T., KHALAF, S., GARCIA, M. & 9 others (1988). Immunoenzymatic assay of Mr 52,000 cathepsin D in 182 breast cancer cytosols: low correlation with other prognostic parameters. *Cancer Res.*, **48**, 462.

MEISSAUER, A., KRAMER, M.D., HOFMANN, M. & 4 others (1991). Urokinase-type and tissue-type plasminogen activators are essential for *in vitro* invasion of human melanoma cells. *Exp. Cell Res.*, **192**, 453.

MIGNATTI, P., ROBBINS, E. & RIFKIN, D.B. (1986). Tumor invasion through the human amniotic membrane: requirement for a proteinase cascade. *Cell*, **47**, 487.

MIRA-Y-LOPEZ, R., OSBORNE, M.P., DEPALO, A.J. & OSSOWSKI, L. (1991). Estradiol modulation of plasminogen activator production in organ cultures of human breast carcinomas: correlation with clinical outcome of anti-estrogen therapy. *Int. J. Cancer*, **47**, 827.

MOSCATELLI, D. & RIFKIN, D.B. (1988). Membrane and matrix localization of proteinases: a common theme in tumor cell invasion and angiogenesis. *Biochem. Biophys. Acta*, **948**, 67.

MULLINS, D.E. & ROHRLICH, S.T. (1983). The role of proteinases in cellular invasiveness. *Biochem. Biophys. Acta*, **695**, 177.

NAKAJIMA, M., IRIMURA, T. & NICHOLSON, G.L. (1987). Basement membrane degradative enzymes and tumor metastasis. *Cancer Bull.*, **39**, 142.

NAITO, S., KINJO, M., NANNO, S., KOHGA, S., OKA, K. & TANAKA, K. (1981). Fibrinolysis-inhibitory activity of cultured human cancer cell lines. *Gann*, **72**, 1.

NEEDHAM, G.K., NICHOLSON, S., ANGUS, B., FARNDON, J.R. & HARRIS, A.L. (1988). Relationship of membrane-bound tissue type and urokinase type plasminogen activators in human breast cancers to estrogen and epidermal growth factor receptors. *Cancer Res.*, **48**, 6603.

O'GRADY, R.L., UPFOLD, L.I. & STEPHENS, R.W. (1981). Rat mammary carcinoma cells secrete active collagenase and activate latent enzyme in the stroma via plasminogen activator. *Int. J. Cancer*, **28**, 509.

O'GRADY, P., LIJNEN, H.R. & DUFFY, M.J. (1985). Multiple forms of plasminogen activator in human breast tumors. *Cancer Res.*, **45**, 6216.

OSSOWSKI, L. & REICH, E. (1983). Antibodies to plasminogen activator inhibit human tumor metastasis. *Cell*, **35**, 611.

OSSOWSKI, L. (1988*a*). Plasminogen activator dependent pathways in the dissemination of human tumor cells in the chick embryo. *Cell*, **52**, 321.

OSSOWSKI, L. (1988*b*). *In vivo* invasion of modified chorioallantoic membrane by tumor cells: the role of cell surface-bound urokinase. *J. Cell Biol.*, **107**, 2437.

PARANJPE, M., ENGEL, L., YOUNG, N. & LIOTTA, L.A. (1980). Activation of human breast carcinoma collagenase through plasminogen activator. *Life Sci.*, **26**, 1223.

QUAX, P.H.A., VAN LEEUWEN, R.T.J., VERSPAGET, H.W. & VERHEIJEN, J.H. (1990). Protein and messenger RNA levels of plasminogen activators and inhibitors analyzed in 22 human tumor cell lines. *Cancer Res.*, **50**, 1488.

REICH, R., THOMPSON, E.W., IWAMOTO, Y. & 4 others (1988). Effects of inhibitors of plasminogen activator, serine proteinases, and collagenase IV on the invasion of basement membranes by metastatic cells. *Cancer Res.*, **48**, 3307.

ROCHEFORT, H., CAVAILLES, V., AUGEREAU, P. & 4 others (1989). Overexpression and hormonal regulation of pro-cathepsin D in mammary and endometrial cancer. *J. Steroid Biochem.*, **34**, 177.

ROCHEFORT, H., CAPONY, F. & GARCIA, M. (1990). Cathepsin D: a protease involved in breast cancer metastasis. *Cancer Metastasis Rev.*, **9**, 321.

ROMAIN, S., MURACCIOLE, X., VARETTE, I., BRESSAC, C., BRANDONE, H. & MARTIN, P.M. (1990). La cathepsine-D: un facteur pronostique indépendant dans le cancer du sein. *Bull. Cancer*, **77**, 439.

SAITO, K., NAGASHIMA, M., IWATA, M. & 4 others (1990). The concentration of tissue plasminogen activator and urokinase in plasma and tissues of patients with ovarian and uterine tumors. *Thromb. Res.*, **58**, 355.

SAPPINO, A.P., BUSSO, N., BELIN, D. & VASSALI, J.D. (1987). Increase of urokinase-type plasminogen activator gene expression in human lung and breast carcinomas. *Cancer Res.*, **47**, 4043.

SCHLECHTE, W., MURANO, G. & BOYD, D. (1989). Examination of the role of the urokinase receptor in human colon cancer mediated laminin degradation. *Cancer Res.*, **49**, 6064.

SCHLEEF, R.R., WAGNER, N.V. & LOSKUTOFF, D.J. (1988). Detection of both type 1 and type 2 plasminogen activator inhibitors in human cells. *J. Cell. Physiol.*, **134**, 269.

SLOANE, B.F., SADLER, J.G., EVENS, C. & 4 others (1984). Cathepsin B-like cysteine proteinases and tumor metastasis. *Cancer Bull.*, **36**, 196.

SLOANE, B.F., MOIN, K., KREPELA, E. & ROZHIN, J. (1990). Cathepsin B and its endogenous inhibitors: the role in tumor malignancy. *Cancer Metastasis Rev.*, **9**, 333.

SPYRATOS, F., BROUILLET, J.P., DEFRENNE, A. & 7 others (1989). Cathepsin D: an independent prognostic factor for metastasis of breast cancer. *Lancet*, **ii**, 1115.

STETLER-STEVENSON, W.G. (1990). Type IV collagenases in tumor invasion and metastasis. *Cancer Metastasis Rev.*, **9**, 289.

TANDON, A.K., CLARK, G.M., CHAMNESS, G.C., CHIRGWIN, J.M. & MCGUIRE, W.L. (1990). Cathepsin D and prognosis in breast cancer. *N. Engl. J. Med.*, **322**, 297.

TESTA, J.E. & QUIGLEY, J.P. (1990). The role of urokinase-type plasminogen activator in aggressive tumor cell behavior. *Cancer Metastasis Rev.*, **9**, 353.

THORPE, S.M., ROCHEFORT, H., GARCIA, M. & 7 others (1989). Association between high concentrations of Mr 52,000 cathepsin D and poor prognosis in primary human breast cancer. *Cancer Res.*, **49**, 6008.

TISSOT, J.D., HAUERT, J. & BACHMANN, F. (1984). Characterization of plasminogen activators from normal human breast and colon and from breast and colon carcinomas. *Int. J. Cancer*, **34**, 295.

TRYGGVASON, K., HÖYHTYÄ, M. & SALO, T. (1987). Proteolytic degradation of extracellular matrix in tumor invasion. *Biochim. Biophys. Acta*, **907**, 191.

TSUBOI, R. & RIFKIN, D.B. (1990). Bimodal relationship between invasion of the amniotic membrane and plasminogen activator activity. *Int. J. Cancer*, **46**, 56.

WILSON, E.L. & FRANCIS, G.E. (1987). Differentiation-linked secretion of urokinase and tissue plasminogen activator by normal human hemopoietic cells. *J. Exp. Med.*, **165**, 1609.

WOHLWEND, A., BELIN, D. & VASSALI, J.D. (1987). Plasminogen activator-specific inhibitors in mouse macrophages: *in vivo* and *in vitro* modulation of their synthesis and secretion. *J. Immunol.*, **139**, 1278.

YAGEL, S., KHOKHA, R., DENHART, D.T., KERBEL, R.S., PARHAR, R.S. & LALA, P.K. (1989). Mechanisms of cellular invasiveness: a comparison of amnion invasion *in vitro* and metastatic behavior *in vivo*. *J. Natl Cancer Inst.*, **81**, 768.

YU, H. & SCHULTZ, R.M. (1990). Relationship between secreted urokinase plasminogen activator activity and metastatic potential in murine B16 cells transfected with human urokinase sense and antisense genes. *Cancer Res.*, **50**, 7623.

ZUCKER, S., LYSIK, R.M., RAMAMURTHY, N.S., GOLUB, L.M., WIEMAN, J.M. & WILKIE, D.P. (1985). Diversity of melanoma plasma membrane proteinases: inhibition of collagenolytic and cytolytic activities by minocycline. *J. Natl Cancer Inst.*, **75**, 517.